# Climate Change Impact on Human-Rodent Interfaces: Modeling Junin Virus Reservoir Shifts

**DOI:** 10.1007/s10393-025-01723-z

**Published:** 2025-06-27

**Authors:** Nuri Flores-Pérez, Pranav Kulkarni, Marcela Uhart, Pranav S. Pandit

**Affiliations:** 1 Department of Population Health and Reproduction, School of Veterinary Medicine, University of California, Davis, One Shields Avenue, Davis, CA 95616 USA; 2 School of Veterinary Medicine, Karen C. Drayer Wildlife Health Center, University of California, Davis, CA USA

**Keywords:** species distribution model, arenaviruses, *Calomys musculinus*, machine learning, argentine hemorrhagic fever, drylands vesper mouse, climate change

## Abstract

The drylands vesper mouse (*Calomys musculinus)* is the primary host for *Junin mammarenavirus* (JUNV), the etiological agent of Argentine hemorrhagic fever in humans. We assessed the potential distribution of *C. musculinus* and identified disease transmission hotspots under current climatic conditions and projected future scenarios, including severe (Representative Concentration Pathway 8.5) and intermediate (Representative Concentration Pathway 4.5) climate change scenarios in 2050 and 2070. Utilizing tree-based machine learning algorithms, we modeled *C. musculinus* distribution by incorporating bioclimatic and landscape predictors. The model showed strong performance, achieving *F*-scores between 80.22 and 83.09%. Key predictors indicated that *C. musculinus* prefers warm temperatures, moderate annual precipitation, low precipitation variability, and low pasture coverage. Under the severe climate change scenario, suitable areas for the rodent and hotspots for potential disease decreased. The intermediate scenario showed an expansion in *C. musculinus* distribution alongside increased potential hotspot zones. Despite the complexity of ecological systems and the limitations of the model, our findings offer a framework for preventive measures and ecological studies in regions prone to the expansion of *C. musculinus* and in hotspots for disease transmission driven by climate change.

**Supplementary Information:**

The online version contains supplementary material available at 10.1007/s10393-025-01723-z.

## Introduction and Purpose

Accelerating climate change is expected to increase the frequency of heatwaves, droughts, and extreme precipitation over the next 2 decades (IPCC 2023). These climate change effects could influence the ecology of zoonotic diseases by altering host and vector distributions, abundance and contact rates with humans. For example, outbreaks of Hantavirus pulmonary syndrome in South and North America have been linked to an increase in food availability for peri-domestic rodents after intense rainfall and flooding (Dash et al. 2021; Rodríguez-Morales and Delgado-López 2012). Similarly, habitat disruptions like wildfires and droughts have been associated with wildlife movements that precede zoonotic spillover events (Mora et al. 2022). Human migration and urbanization driven by climate change may also influence disease risk as areas with high human population density are predictive of infectious disease emergence (Eskew and Olival 2018; Jones et al. 2008).

The ecological changes generated by climate change may be relevant for rodent-borne diseases, such as Argentine hemorrhagic fever (AHF), caused by the *Junin mammarenavirus* (JUNV). This disease is endemic to the humid pampas in Argentina, a densely populated area at the core of the largest agro-industrial complex in the country (Briggiler et al. 2015; Enria et al. 1998). In severe cases of AHF, patients develop hemorrhagic and neurological complications, with a fatality rate of 20% (Gallo et al. 2022). AHF was first reported in 1955 among agricultural workers (Carballal et al. 1988). It is believed that the outbreak was due to land use changes and occupational exposure via agricultural activities (Enria et al. 2008). Since its emergence, a constant and progressive extension of the endemic area has been observed (Polop et al 2007). In the last 10 years, the disease has expanded to northeastern Argentina, with recent reemergence in areas that had not had cases for 15–20 years (Calderón et al. 2022). In 1992, the Candid#1 vaccine was applied to at-risk populations, causing a decrease in AHF incidence (Briggiler et al. 2015; Bausch and Mills 2014). However, in the following years, changes in the risk patterns were observed, with increased cases among women, children under 15 years old, and people who work in non-rural jobs and/ or reside in urban zones (Briggiler et al. 2015). Between January 1 and July 14, 2024, there were 33 confirmed cases of AHF, including six fatalities, reflecting an increase compared to the past eight years (MSAL 2024).

The main reservoir of AHF is *C. musculinus* (*Cricetidae: Sigmodontinae*). This rodent is found in Argentina, Bolivia, and Paraguay. It occupies a wide variety of habitats, like natural grasslands, shrub steppes, crop field borders, and human-disturbed environments, such as wastelands, railroads, and urban garbage dumps (Provensal et al. 2019; Mills et al. 1992). Its abundance changes seasonally, driven by natural climatic variations and land-use practices that affect resource availability (Sommaro et al. 2022). Corn crops and land-surface temperature have been identified as key predictors of increased abundance in this species, while field borders consistently provide habitat, food, and shelter throughout the year (Simone et al. 2012).

Understanding how climate change may alter the distribution of *C. musculinus* is therefore critical for anticipating future AHF risk. Species distribution models (SDM) estimate suitable habitats by correlating species occurrences with measured or estimated environmental variables (Miller 2010; Phillips et al. 2004). These models rely on several key assumptions: environmental conditions reflect current species distribution, species are in equilibrium with their environment, environmental predictors capture limiting factors for species distributions, pseudo-absences represent unsuitable habitats, and species can disperse to all climatically suitable areas (Elith and Leathwick 2009; Jeschke and Strayer 2008). While SDMs were originally used to explore ecological drivers of species distributions, they now prioritize predictive applications such as forecasting climate-driven range shifts, identifying conservation or invasive species hotspots, and reconstructing historical distributions. Despite this shift toward practical applications, ecological insight remains vital for selecting biologically meaningful predictors and interpreting model uncertainty.

In South America, previous studies have used maximum likelihood (Porcasi et al. 2005) and cellular automaton models (Musso et al. 2011) to predict the potential distribution of *C. musculinus* using bioclimatic and landscape data. Expanding these approaches under current and future climate scenarios can improve surveillance systems and guide more effective public health interventions. Furthermore, modeling offers an effective alternative to extensive, time consuming and resource-intensive field studies. In this study, we developed a SDM for *C. musculinus* based on bioclimatic and landscape variables. We incorporated machine learning (ML) classifiers and considered future climate change scenarios to anticipate potential shifts in the rodent distribution to address three objectives: (i) to evaluate the importance and impact of different bioclimatic and landscape variables in the prediction of *C. musculinus* range shifts, (ii) to assess these geographic changes under current and future climate change scenarios, and (iii) to identify hotspots for potential disease transmission.

## Methods

**Figure 1:**
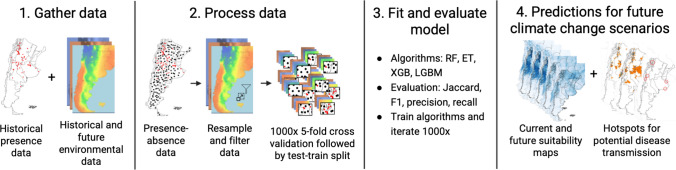
Methodological workflow for building the species distribution model of *Calomys musculinus* using four machine learning algorithms (Random Forest – RF, Extra Trees – ET, Extreme Gradient Boosting – XGB, and Light Gradient-Boosting – LGBM).

To provide an overview of the methodological framework, Figure 1 summarizes the steps of the process.

### Study Area

The study focused on Argentina to assess the current and future distribution for *C. musculinus*. Argentina covers 2,780,400 km^2^ with a 3694 km north–south span. Agriculture is a major economic activity, consisting mainly of monocultures of corn, soybean, sunflower, and wheat (Sommaro et al. 2022). The human population is predicted to grow from the current 46 to 73.4 million by 2100 (INDEC 2024; González 2015).

Argentina is divided into 18 ecoregions, which are territories with relatively uniform or recurrent environmental conditions (Fig. S1; Burkart et al. 1999). There has been a progressive extension of the AHF endemic region into the north-central area of the Pampas ecoregion. This expansion poses a potential risk to approximately 5 million people (Kumar et al. 2023).

### Species Occurrence Data

Presence data for *C. musculinus* was retrieved from the Global Biodiversity Information Facility (GBIF; https://doi.org/10.15468/dd.v4aq9c), limiting the occurrences to the bounds of Argentina. To align the presence data period closely with the bioclimatic and landscape variables period, we constrained the dataset to records spanning from 1990 to the last available year of GBIF records. After deleting duplicates and missing values, the refined dataset consisted of 70 observations from 1990 to 2021.

### Predictor Bioclimatic and Landscape Variables

Based on relevance to *C. musculinu*s ecology and data availability, we selected 26 bioclimatic and landscape predictors (Table S1), including 19 bioclimatic variables from WorldClim (Fick and Hijmans 2017), five land use variables from Harmonized Global Land Use (Chini et al. 2014), and one elevation variable from the Consortium for Spatial Information (Jarvis et al. 2008). As the feature importance assessment (explained below) can neglect the importance of correlated features (Petelin et al. 2023), we utilized the Pearson correlation coefficient to exclude highly correlated variables (r ≥ 0.7) and built our model with only the features with low spatial correlation (Table 1).

**Table 1:** Bioclimatic and Landscape Variables used to Build *C. musculinus* Species Distribution Model.

Category	Description	Code
Bioclimatic variables (Fick and Hijmans 2017)	Annual mean temperature	Bioclim 1
	Isothermality	Bioclim 3
	Annual temperature range	Bioclim 7
	Mean temperature of driest quarter	Bioclim 9
	Annual precipitation	Bioclim 12
	Precipitation seasonality	Bioclim 15
Land use variables (Chini et al. 2014)	Percentage of cropland cover	Cropland
	Percentage of primary land cover	Primary land
	Percentage of secondary land cover	Secondary land
	Percentage of pasture cover	Pasture
	Percentage of urban land cover	Urban land
Digital elevation model (Jarvis et al. 2008)	Elevation	DEM

We used two Representative Concentration Pathways (RCPs) from the Coupled Model Intercomparison Project Phase 5 (CMIP5; Taylor et al. 2012)—RCP4.5 (intermediate greenhouse gas emissions) and RCP8.5 (high greenhouse gas emissions)—to calculate the predicted distribution in future climate change scenarios for the years 2050 and 2070. RCPs represent a range of possible future radiative forcing levels based on emissions of greenhouse gases and aerosols (Hibbard et al. 2011).

All raster variables were sourced to GeoTiff files and resampled to align with the dimensions (597 × 999), resolution (0.042°), and projection (WGS84) of the bioclimatic variables. This ensured consistent spatial representation across all raster files, facilitating accurate integration for the analysis.

### Species Distribution Model

We generated 1000 training datasets, each with 70 presence points paired with 70 randomly generated pseudo-absences points, to ensure robustness and reliability in the modeling process and results. Pseudo-absences were created using the *randomPoints()* function in R software version 4.2.2. A 1:1 ratio of presence to pseudo-absences yielded the highest model accuracy, as described by Barbet-Massin et al. (2012).

We employed an ensemble approach utilizing four tree-based machine learning (ML) algorithms: random forest (RF), extra trees (ET), extreme gradient boosting (XGB), and light gradient-boosting (LGBM). The model was trained in Python version 3.10.9 using scikit-learn library (Pedregosa et al. 2011) for RF and ET, XGBoost library (Chen and Guestrin 2016) for XGB, and LightGBM library (Ke et al. 2017) for LGBM. Each algorithm was trained using its respective default hyperparameters as implemented in the standard libraries. Common hyperparameters across these algorithms include the number of trees, maximum tree depth, learning rate (for boosting methods), and minimum samples required to split a node or define a leaf. Detailed parameter settings are available in the official documentation and summarized in S1 Appendix. Predictions for current and projected scenarios were generated using the *impute* function from the PyImpute library in Python 3.8 (Perry et al., 2014). Each classifier was trained 1000 times and predictions were averaged to generate habitat suitability maps for *C. musculinus*. Performance was evaluated using fivefold cross-validation with four metrics: (i) *F*-score, (ii) recall, (iii) precision, and (iv) Jaccard index. In addition, we performed external validation using an independent test dataset to further assess the robustness and generalizability of our model. To ensure a sufficient sample size for both model training and validation, we implemented a 25:75% split for testing and training datasets, evaluating performance based on (i) precision and (ii) recall for predicted presences.

For variable importance, we used Mean decreased impurity (MDI) for the RF and ET (Chavent et al. 2021), and Gain Feature Importance (Shi et al. 2019) for XGB and LGBM. For each ML classifier, we averaged the 1000 simulation results and generated boxplots to summarize findings. Similarly for each iteration, we generated partial dependence plots (PDPs) to understand how input variables influence the target response (Hastie et al. 2009). We averaged the 1000 simulation results of each classifier to generate final PDPs. Distribution of *C. musculinus* was generated by averaging predictions across all four algorithms. We compared current and future predictions to assess potential shifts in *C. musculinus* distribution under climate change using the following formula:

Predicted change of distribution = Projected suitability − Current suitability.

We applied this formula to two future climate scenarios, RCP4.5 (intermediate greenhouse gas emissions) and RCP 8.5 (high greenhouse gas emissions), for 2050 and 2070. For each scenario and year, we identified areas where species distribution is likely to expand (positive values), contract (negative values), or remain stable (values close to zero).

### Hotspots for Potential Disease Transmission

We obtained human population density data for Argentina from the Socioeconomic Data and Applications Center (Jones and O’Neill 2016). This information is based on the CMIP—Shared Socioeconomic Pathways (SSP 2 and SSP 5) which are analogous to the RCP 4.5 and RCP 8.5 scenarios, consisting of global population data for the base year 2000 and projections at ten-year intervals for 2010–2100 (Jones and O’Neill 2016).

We set a suitability threshold of 60% or greater to designate the presence of *C. musculinus*, prioritizing the reduction of false positives and minimizing the risk of overestimating habitat suitability. Regarding the human population density, we established three tiers: (i) human population density greater than 1000, (ii) 10,000 and (iii) 100,000. These thresholds were chosen to capture areas with varying human population densities, ensuring that hotspots are identified in regions with potential human-rodent overlap and subsequent disease transmission. We generated a raster layer to represent areas that satisfied both criteria, indicating regions where the threshold of *C. musculinus* presence suitability overlapped with the human population density thresholds. These areas were classified as hotspots, delineating zones of potential disease transmission.

## Results

### Model Comparison and Evaluation

After omitting 13 highly correlated bioclim variables (*r* ≥ 0.7) (Fig. S2) our model included annual mean temperature, isothermality, annual temperature range, mean temperature of driest quarter, annual precipitation, and precipitation seasonality, all land use variables, and elevation (Table 1).

Table 2 shows that extra trees (ET) and random forest (RF) had the highest *F*-scores and Jaccard indices, with minimal differences between cross-validation and test set precision and recall. Extreme gradient boosting (XGB) and light gradient-boosting (LGBM) had slightly lower *F*-scores and Jaccard indices, but still achieved high precision and recall values that were similar between cross-validation and test set validation.

**Table 2:** Performance Metrics of the Machine Learning (ML) Classifiers Used for Predicting *C. musculinus* Distribution.

ML algorithm	Cross-validation metrics (five-fold cross-validation)				Test set validation metrics (25% test set)	
	Jaccard index (±) %	*F*-score (±) %	Precision (±) %	Recall (±)%	Precision (±) %	Recall (±)%
Random forest	70.35(16.85)	82.54(13.09)	80.54(18.55)	84.54(18.40)	79.91(6.03)	85.24(4.68)
Extra Trees	71.16(16.62)	83.09(12.87)	80.99(18.52)	85.30(17.71)	79.5(5.96)	86.43(4.77)
Extreme gradient boosting	67.50(17.26)	80.54(13.56)	78.75(19.14)	82.41(19.16)	80.12(5.45)	83.93(5.07)
Light gradient boosting	67.07(17.35)	80.22(13.66)	78.19(19 18)	82.36(19.40)	80.24(6 62)	84.84(4.95)

### Importance of Bioclimatic and Landscape Variables

**Figure 2:**
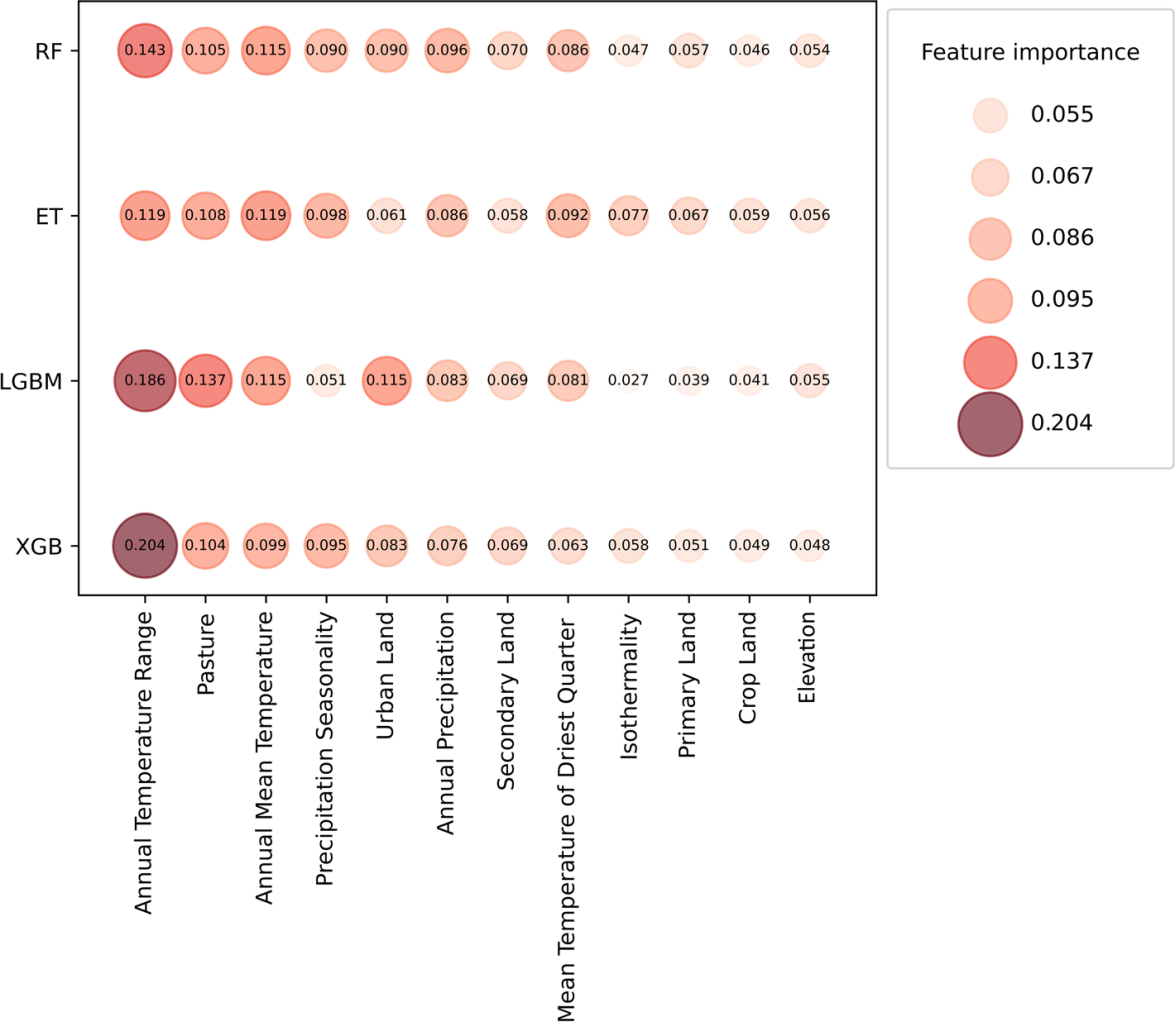
Importance of bioclimatic and landscape variables used for predicting *C. musculinus* distribution. The influence of bioclimatic and landscape variables is shown across the four classifiers used for predicting AHF reservoir distribution: Random Forest (RF), Extra Trees (ET), Extreme Gradient Boosting (XGB), and Light Gradient-Boosting (LGBM). *Note* Bigger circles and higher values indicate a greater importance.

Figure 2 shows the influence of each variable across the four algorithms, while a summary of the 1000 simulation results of feature importance can be found in the supplementary material (Fig. S3). For RF, the most important feature was the annual temperature range, followed by annual mean temperature and pasture. Similarly, ET ranked annual mean temperature higher, followed by annual temperature range and pasture. For XGB and LGBM, the most important feature was annual temperature range, followed by pasture. XGB classifier had annual mean temperature in the third place, while LGBM had urban land.

Figure S4 shows partial dependence plots for key bioclimatic and landscape variables. The species suitability increased when the annual temperature range increased but decreased around 30 °C according to the ET classifier. In the RF, XGB and LGBM, the response started weak, increasing after 20 °C and decreasing around 30 °C. For annual mean temperature, in all the classifiers, after 5 °C there was increasing suitability when the temperature increased, after that, there was a decrease around 17 °C. As for annual precipitation, there was a weak response until 700 mm of total annual precipitation, where partial dependence of the species started to decrease as the precipitation increased. On the other hand, the suitability of the rodent increased as the precipitation seasonality increased, but the response appeared weak at around 80% of precipitation variability, except in the ET classifier, which showed a decrease in suitability as precipitation seasonality increased.

For the RF, XGB, and LGBM classifiers, the partial dependence of *C. musculinus* presence increased with increasing pasture coverage until 10% coverage. The partial dependence of the rodent decreased by around 20% for all ML models, becoming weak after 50%. In particular, the ET classifier showed a constant decrease in partial dependence after 20% of pasture cover. For urban land, the rodent's presence increased with increasing urban land coverage but became weak over 0.5% of coverage.

### Distribution of *C. musculinus* under Current and Climate Change Scenarios

**Figure 3:**
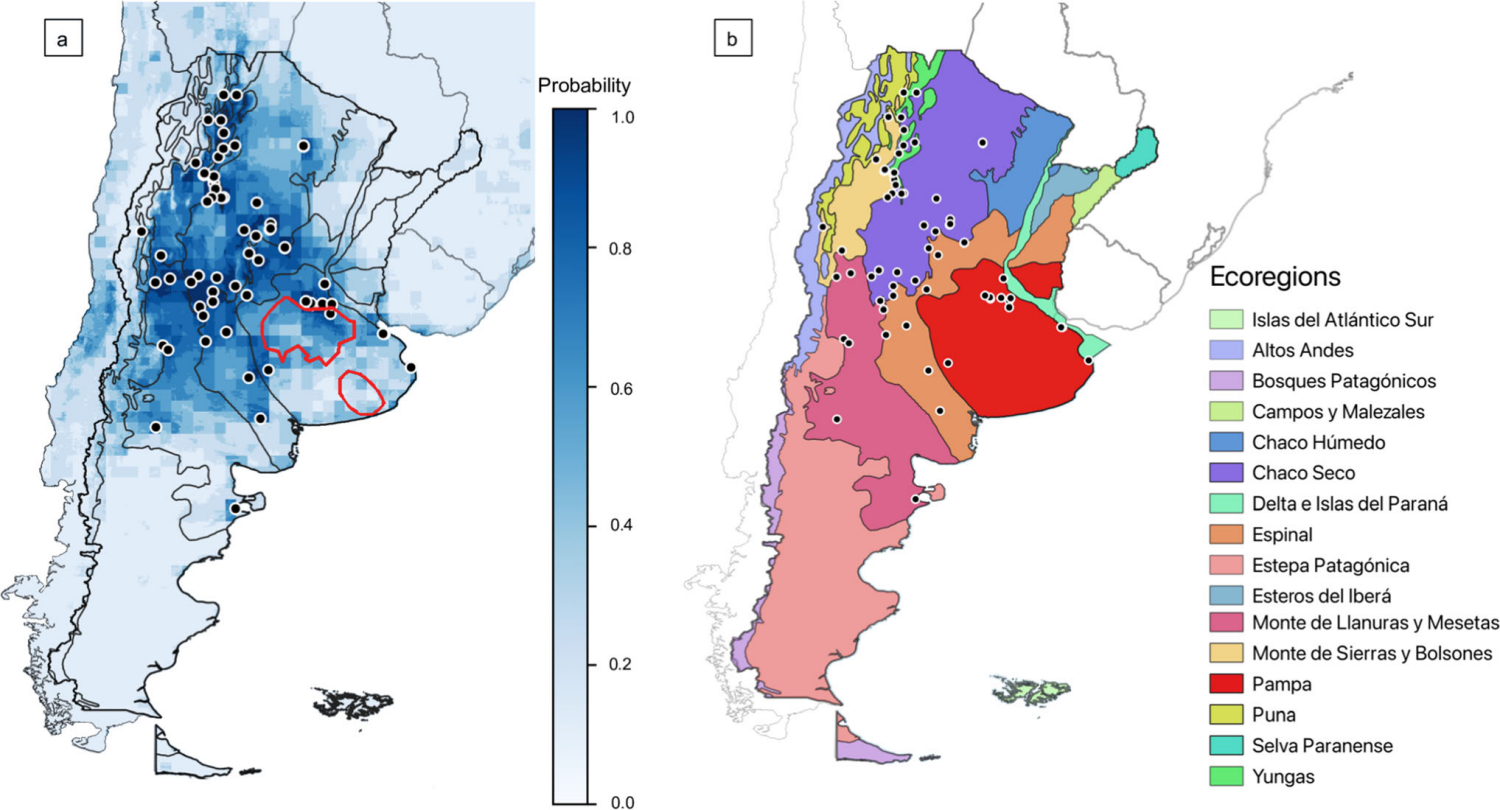
Predicted distribution of *C. musculinus* in the ecoregions of Argentina under current bioclimatic and landscape conditions. **a** Current predicted distribution of *C. musculinus* with higher values and darker blue colors indicating a greater suitability. A red outline marks the endemic area of Argentine hemorrhagic fever. **b** The ecoregions of Argentina as defined in Methods section, overlaid with *C. musculinus* occurrence data from GBIF (black dots).

The current predicted distribution matched *C. musculinus* occurrence data from GBIF and showed high suitability in Pampas, Espinal, Monte de Llanuras y Mesetas, Chaco seco, Monte de Sierras y Bolsones, and Yungas ecoregions (Fig. 3).

**Figure 4:**
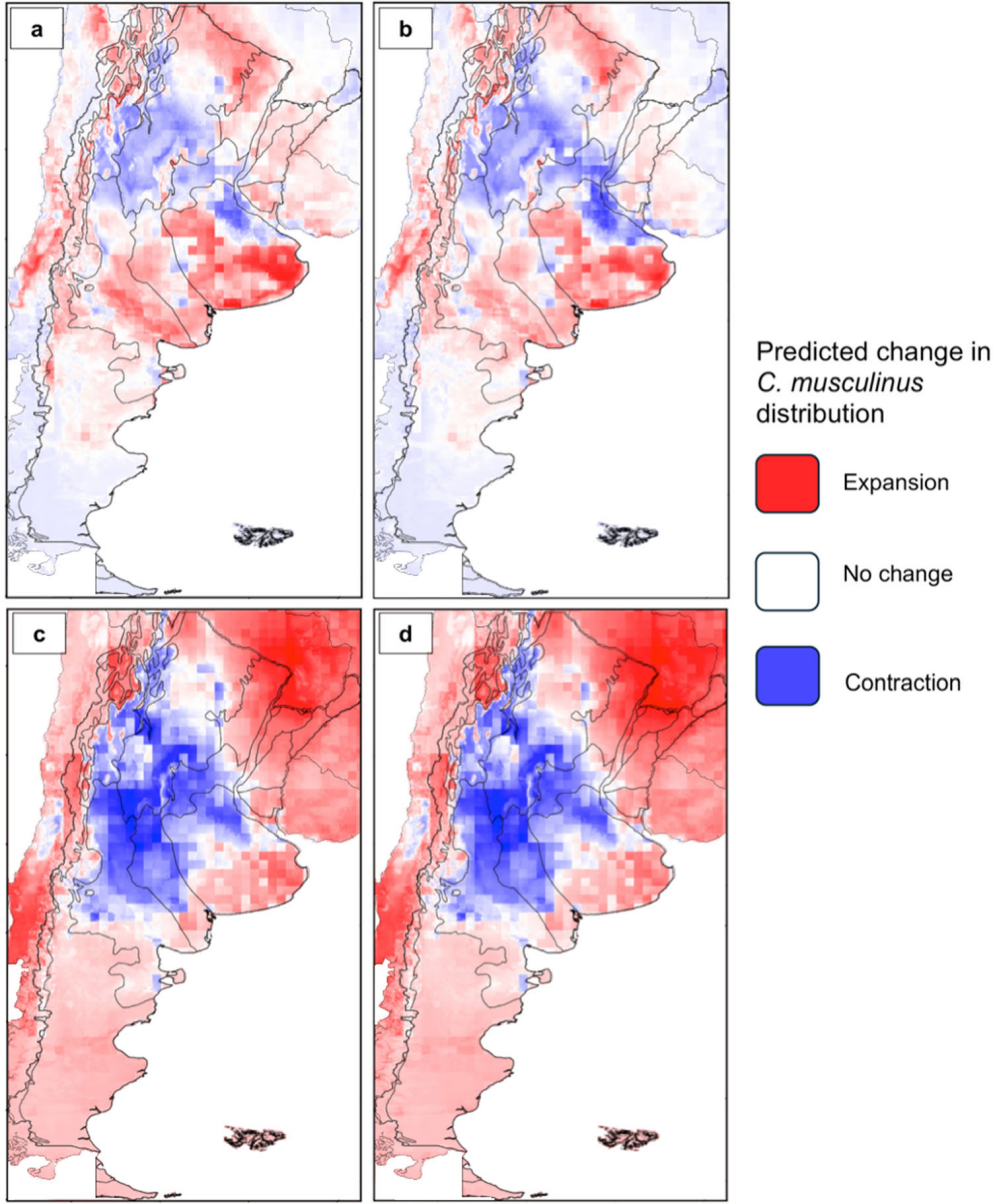
Predicted change in *C. musculinus* distribution between current and future climate change scenarios. Expansion (red), contraction (blue), and areas with no predicted change (white) between current and future climate change scenarios and years: **a** RCP4.5 for year 2050, **b** RCP4.5 for year 2070, **c** RCP 8.5 for year 2050, and **d** RCP 8.5 for year 2070.

Figure 4 displays predicted change in *C. musculinus* distribution between the current conditions and under the RCP4.5 and RCP8.5 scenarios. Under RCP4.5 for 2050 and 2070, the distribution contracted in northeast Pampas, northern Espinal, southern Chaco Seco and Monte de Sierras y Bolsones, northern Monte de Llanuras y Mesetas, and most of the Yungas and Selva Paranaense. The other ecoregions showed expansion, and there were a few areas with no change, like southern Estepa Patagónica. The predicted areas of expansion or contraction in both scenarios are similar, but in 2070 they showed a decrease in expansion and an increase in contraction.

In the RCP8.5 scenario for 2050 and 2070, *C. musculinus* distribution expanded in east Espinal, Esteros del Iberá, Campos y Malezales, Selva Paranaense, Chaco Húmedo, Bosques Patagónicos, Altos Andes, Puna, southern Estepa Patagónica, southern Pampas, northern Chaco seco, southern Espinal, and northern Yungas. The rest of the ecoregions showed contraction. Expansion increased between 2050 and 2070, especially in the north and south, while contraction increased in Monte de Llanuras y Mesetas and Monte de Sierras y Bolsones. Between RCP4.5 and RCP8.5 for both 2050 and 2070, the predicted contraction in the RCP8.5 scenario extended and increased in Monte de Llanuras y Mesetas, Monte de Sierras y Bolsones, Espinal, western Pampas and most areas of Chaco seco. Moreover, the predicted expansion increased in northern and southern Argentina, reaching neighboring Chile, Paraguay, Brazil, and Bolivia.

### Hotspots for Potential Disease Transmission

**Figure 5:**
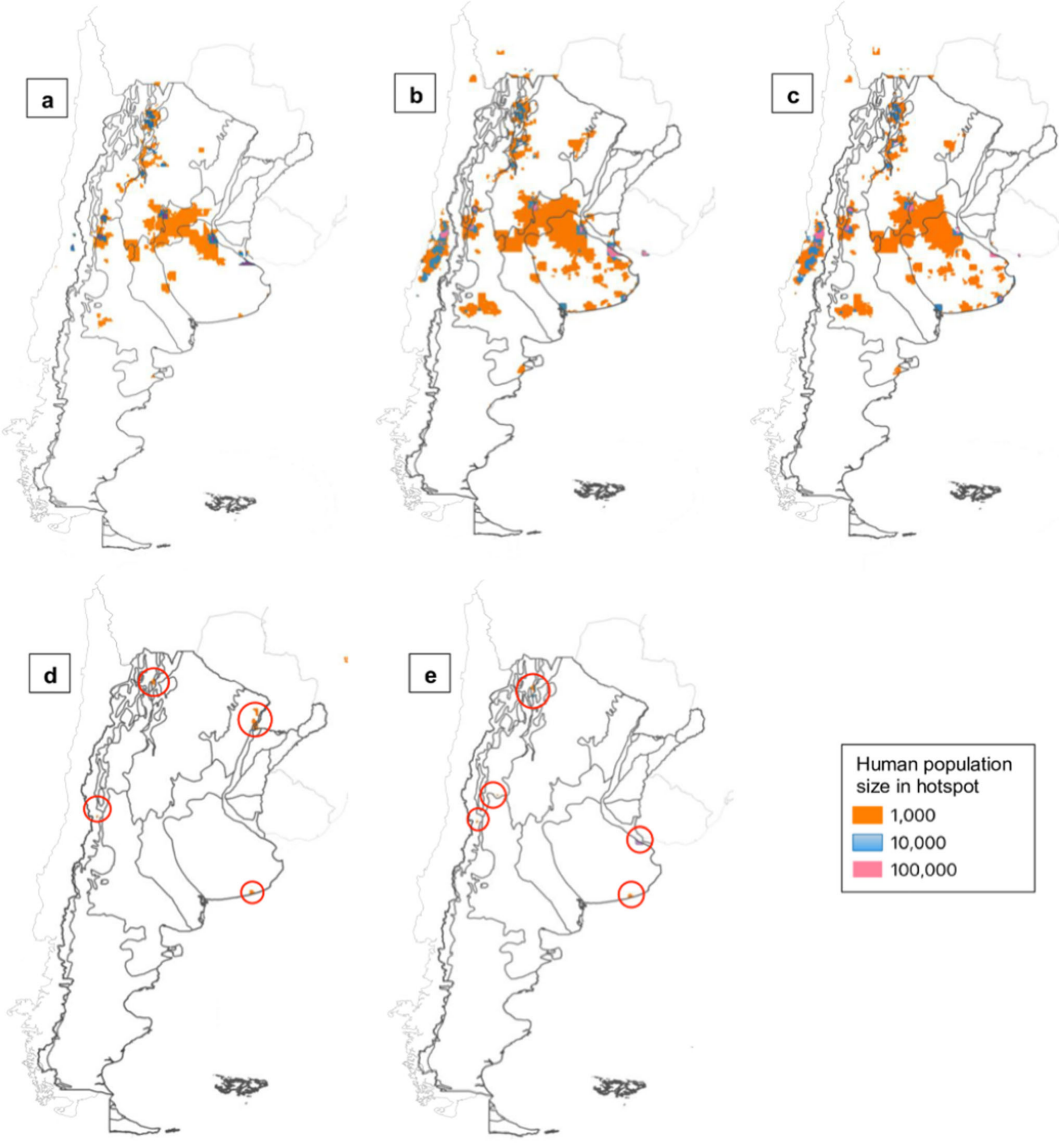
Hotspots for potential AHF disease transmission for current and future climate change scenarios. Hotspots are displayed according to three total human population density thresholds (1000, 10,000 and 100,000) under: **a** current conditions, **b** RCP4.5 scenario for year 2050, **c** RCP4.5 scenario for year 2070, **d** RCP8.5 scenario for year 2050, and **e** RCP8.5 scenario for year 2070. *Note* Red circles point out the predicted hotspots in the RCP8.5 scenario.

Under current conditions and both climate change scenarios, hotspots decreased as human population density increased (Fig. 5). Current hotspots were concentrated in Pampas, Espinal, Chaco Seco, Yungas, Monte de Llanuras y Mesetas, and Monte de Sierras y Bolsones. In the RCP4.5 scenario, hotspots were present in the same ecoregions but their size increased. In 2070, the number of hotspots decreased compared to 2050. In the RCP8.5 scenario for the year 2050, hotspots were in Pampas, Altos Andes, Monte de Sierras y Bolsones, Yungas and Puna. The year 2070 in the RCP8.5 scenario displayed hotspots in Pampas, Altos Andes, Puna, and Chaco Húmedo. For this latter year and scenario, there were no hotspots for the 100,000 human density population threshold.

## Discussion

Our findings highlight the impact of climate change on the transmission risk of AHF, a historically neglected disease yet severe public health concern. Unlike vector-borne diseases where climate directly affects mosquito ecology, our study demonstrates that AHF transmission is indirectly influenced by climate-driven shifts in the distribution of *C. musculinus*. By modeling its distribution under various climate change scenarios, we offer a framework for addressing future public health challenges. Our findings underscore the complex interplay between climate change, rodent ecology, and human health, highlighting the need for adaptive strategies in response to changing disease dynamics.

Our research revealed that *C. musculinus* prefers environments with warm temperatures, moderate annual precipitation, low precipitation variability, and low pasture coverage. Our model complements previous studies (Musso et al. 2011; Porcasi et al. 2005), showing how climate change might affect *C. musculinus* distribution and highlighting areas of potential disease transmission to humans. For current climate conditions, our model aligned with GBIF records and the recent assessment of the distribution of the rodent (SAyDS and SAREM 2019). It also partially overlapped with the distribution estimated by Porcasi et al. (2005), as our predictions showed a more restricted distribution in Estepa Patagónica and south of Monte de Llanuras y Mesetas. Notably, predicted suitability under current conditions also matched areas of the endemic zone of AHF in the Pampas ecoregion, and under the RCP4.5 scenario, there is an increase and expansion in suitability within the endemic zone. These suitable areas within the endemic zone, as well as surrounding areas, warrant close monitoring and preventative measures.

The effects of climate change on AHF host distribution and transmission risk varied by climate change scenario. Both the intermediate (RCP4.5) and extreme (RCP8.5) scenarios showed areas of expansion and contraction, but the RCP4.5 scenario presented more and larger hotspots for potential disease transmission compared to the RCP8.5 scenario and current climate conditions. In a broader context, García-Peña et al. (2021) identified increased rodent zoonotic hazard under the Shared Socioeconomic Pathway 5 scenario (analogous to RCP8.5) for Northern South America and southern Argentina, which differs from the reduced hotspot areas we observed under RCP8.5 scenario. Similarly, Klitting et al. (2022) projected that under RCP6.0 and RCP8.5, *Lassa virus* could expand into Central and East Africa, in regions where *Mastomys natalensis* is expected to be present. Differences across studies likely arise from variation in geographic scope, reservoir species, and modeling focus. While García-Peña et al. assessed zoonotic hazard across multiple rodent species and Klitting et al. modeled a virus-host system, our work concentrated on a single host species. These findings highlight that climate change can reshape zoonotic risk in multiple and complex ways, emphasizing the importance of integrating broad-scale, virus-specific and species-specific approaches to guide targeted public health interventions.

Annual temperature range, annual mean temperature, annual precipitation, precipitation seasonality, pasture, and urban land usage were important factors in improving ML classifier performance. The results suggested that *C. musculinus* prefers annual mean temperatures between 10 and 17 °C, and annual temperature ranges of 22–27 °C. This coincides with Simone et al. (2012) who documented a positive correlation between rodent abundance and land-surface temperatures during the coldest periods. An annual precipitation greater than 800 mm of rainfall and precipitation seasonality greater than 70% may decrease the habitat suitability for the rodent. These results align with studies showing that spring and summer precipitation favors population growth by increasing plant resources but causes higher mortality in winter (Tapia-Ramírez et al. 2022; Fraschina et al. 2012).

Habitat suitability for *C. musculinus* decreased when pasture coverage reached 20%, possibly due to displacement by specialist species like Azara’s grass mouse (*Akodon azarae)* (Gomez et al. 2015). Furthermore, the urban land feature improved LGBM and XGB classifier performance. The partial dependence plot showed that species presence increased as urban land usage increased but flattened after 0.5% coverage. However, the initial increase could indicate that peri-urban zones might be suitable for the species, aligning with Castillo et al. (2003), who observed abundance in unoccupied areas, garbage dumps, and railway and stream edges within the city of Río Cuarto, Córdoba, Argentina. Nevertheless, Chiappero et al. (2011) suggested that these urban rodent populations might have been native to the area, but subsequently encircled by the expansion of the city. If the encircling of rodent habitats by urbanization continues, it becomes necessary to examine the potential implications for public health. Overall, the predictive power of our model depends on the validity of extrapolation, highlighting the need for cautious interpretation.

While previous studies have linked *C. musculinus* presence to crops and crop borders (Simone et al. 2012; Busch et al. 2000), this variable was less important in our model. One possible explanation is that rodent occurrence data was recorded more frequently in pasture lands rather than croplands, thereby adding sampling bias. Despite this, climate change may lead to modifications in agricultural practices (Mall et al. 2017). In Argentina, warmer and wetter conditions could cause an extension of croplands towards the southern and western Pampas ecoregion (Barros et al. 2015). Rising temperatures may favor certain crops, like soybean over corn and wheat crops (Barros et al. 2015). Studying rodent community responses to these changes could offer insights into the *C. musculinus* potential distribution and AHF risk.

At the time of our study, crop cover data for ecoregions of Argentina was unavailable. Previous studies indicated that corn crops and stubbles positively predict *C. musculinus* abundance (Simone et al. 2012). Therefore, incorporating crop type data could enhance model predictions. Finally, multispecies modeling could improve accuracy by accounting for how other species affect the dynamics and distribution of *C. musculinus* (Simone et al. 2010; Busch et al. 2000).

While interpreting the inferred range shifts, some limitations must be considered. Some species may adapt in ways models do not capture or face barriers to reaching predicted suitable areas. For example, although our model includes predictions for Islas del Atlántico Sur, these areas are inaccessible to *C. musculinus* due to their geographic isolation. Even if these regions appear to have low habitat suitability, actual migration is not possible unless facilitated by human-mediated transport. Additionally, ecosystem fragmentation can further obstruct dispersal and migration, affecting the ability of the species to move to suitable habitats in response to climate change (Chiappero et al. 2016; Collingham and Huntley 2000).

Due to limited data availability, there was a slight temporal mismatch in the model training data. Updated species occurrence data aligned with the temporal resolution of bioclimatic and landscape variables could strengthen the reliability of the findings. Incorporating other variables (which are currently unavailable for the years under study) could improve predictions.

Managing hotspots of high human-rodent interaction in Argentina requires adaptive strategies to address Junin virus spread amid climate change. In densely populated areas (> 10,000 people/km^2^), regular rodent trapping and testing are crucial for early detection of population changes. Sentinel surveillance sites should be established in expanding hotspots, particularly in the Pampas and Espinal ecoregions, to monitor *C. musculinus* population dynamics. Developing climate-based early warning systems with real-time data could improve predictions of rodent population surges and disease risk. Prevention should focus on rodent-proofing in areas with 0.5–10% urban land coverage.

In rural hotspots, particularly in the Pampas ecoregion where agricultural activities are prevalent, promoting agricultural practices that limit rodent habitats is essential, especially in areas with 10–20% pasture coverage. Ecologically based rodent management (EBRM) practices are valuable in this context, as they aim to reduce rodent populations through environmental-friendly interventions rather than synthetic chemical control. These practices include maintaining low vegetation cover along field borders, promoting crop rotation and post-harvest crop residue grazing, and stimulating predator presence by reducing cover (Ruscoe et al. 2021; Coda et al. 2014; Dalecky et al. 2014; Simone et al. 2012; Piacenza et al. 2011). Implementing these practices, informed by our model predictions and hotspot analysis, can support public health efforts to mitigate AHF transmission risk while also directly protecting the environment, agricultural workers and rural communities.

Addressing the challenges of *C. musculinus* habitat shifts requires consideration of interventions and their effectiveness. Public education campaigns can enhance awareness about JUNV transmission and promote preventative behaviors. Moreover, training health professionals on AHF diagnosis, treatment and prevention and organizing immunization campaigns with the Candid#1 vaccine are crucial. The assessment of pre- and post-campaign surveys and analysis of infection rates could be a tool to ensure these interventions effectively reduce the risk of transmission.

## Conclusion

Our study provides a valuable tool for understanding general trends in the distribution of *C. musculinus* in response to climate change. By identifying potential areas of habitat suitability and the factors that could be influencing these shifts, we offer information for public health interventions targeting AHF prevention. Targeted preventive measures, such as rodent ecological studies and epidemiological surveillance, could be directed toward regions predicted as potential expansion areas for *C. musculinus*. Moreover, human vaccination efforts could also be enhanced to address the risk of shifts in the disease dynamics that are made apparent using our framework, particularly in the identified hotspots for potential disease transmission. Our framework and its findings underscore the role of modeling predictive tools in guiding public health strategies, emphasizing the crucial need for continuous innovation and collaboration to effectively address evolving disease dynamics amidst the challenges posed by climate change.

## Supplementary Information

Below is the link to the electronic supplementary material.

Supplementary file1 (DOCX 19 KB)

Supplementary file2 (DOCX 17 KB)

Supplementary file3 (TIFF 521 KB)

Supplementary file4 (TIFF 1835 KB)

Supplementary file5 (TIF 1803 KB)

Supplementary file6 (TIF 1575 KB)

Supplementary file7 (TIF 2054 KB)

Supplementary file8 (TIF 3382 KB)
