# Supplementary material for: Climate Change Impact on Human-Rodent Interfaces: Modeling Junin Virus Reservoir Shifts
Source: Ecohealth. 2025 Jun 27;22(3):332–45. doi: 10.1007/s10393-025-01723-z (PMC12476445; doi:10.1007/s10393-025-01723-z)
Supplement: Supplementary file 1 — Supplementary file1 (DOCX 19 KB) [file 10393_2025_1723_MOESM1_ESM.docx]

**S1 Appendix. Default hyperparameters used in machine learning algorithms for modeling Calomys musculinus distribution**

**Random Forest (RF)**

- **n_estimators***int=100*
- **criterion** *=”gini”*
- **max_depth=None**
- min_samples_split*=2*
- min_samples_leaf*=1*
- min_weight_fraction_leaf*=0.0*
- max_features*=”sqrt”*
- max_leaf_nodes*=None*
- min_impurity_decrease*=0.0*
- bootstrap*=True*
- oob_score *=False*
- n_job*=None*
- random_state*=None*
- verbose*=0*
- warm_start *=False*
- **class_weight***None*
- ccp_alpha *=0.0*
- max_samples*=None*

**Extra Trees (ET)**

- *n_estimators=100*
- *criterion='gini'*
- *max_depth=None*
- *min_samples_split=2*
- *min_samples_leaf=1*
- *min_weight_fraction_leaf=0.0*
- *max_features='sqrt'*
- *max_leaf_nodes=None*
- *min_impurity_decrease=0.0*
- *bootstrap=False*
- *oob_score=False*
- *n_jobs=None*
- *random_state=None*
- *verbose=0*
- *warm_start=False*
- *class_weight=None*
- *ccp_alpha=0.0*
- *max_samples=None*

**Extreme Gradient Boosting (XGB)**

- objective: 'binary:logistic'
- use_label_encoder: None
- base_score: None
- booster: None
- callbacks: None
- colsample_bylevel: None
- colsample_bynode: None
- colsample_bytree: None
- early_stopping_rounds: None
- enable_categorical: False
- eval_metric: None
- feature_types: None
- gamma: None
- gpu_id: None
- grow_policy: None
- importance_type: None
- interaction_constraints: None
- learning_rate: None
- max_bin: None
- max_cat_threshold: None
- max_cat_to_onehot: None
- max_delta_step: None
- max_depth: None
- max_leaves: None
- min_child_weight: None
- missing: np.nan
- monotone_constraints: None
- n_estimators: 100
- n_jobs: None
- num_parallel_tree: None
- predictor: None
- random_state: None
- reg_alpha: None
- reg_lambda: None
- sampling_method: None
- scale_pos_weight: None
- subsample: None
- tree_method: None
- validate_parameters: None
- verbosity: None

**Light Gradient-Boosting (LGBM)**

- boosting_type: 'gbdt'
- class_weight: None
- colsample_bytree: 1.0
- importance_type: 'split'
- learning_rate: 0.1
- max_depth: -1
- min_child_samples: 20
- min_child_weight: 0.001
- min_split_gain: 0.0
- n_estimators: 100
- n_jobs: -1
- num_leaves: 31
- objective: None
- random_state: None
- reg_alpha: 0.0
- reg_lambda: 0.0
- silent: 'warn'
- subsample: 1.0
- subsample_for_bin: 200000
- subsample_freq: 0
