# Supplementary material for: Climate Change Impact on Human-Rodent Interfaces: Modeling Junin Virus Reservoir Shifts
Source: Ecohealth. 2025 Jun 27;22(3):332–45. doi: 10.1007/s10393-025-01723-z (PMC12476445; doi:10.1007/s10393-025-01723-z)
Supplement: Supplementary file 2 — Supplementary file2 (DOCX 17 KB) [file 10393_2025_1723_MOESM2_ESM.docx]

**S1 Table. Bioclimatic and landscape variables relevant to *C. musculinus* distribution.**

| Variable | Description | Code |
| --- | --- | --- |
| Bioclimatic variables (Fick and Hijmans, 2017) | Annual mean temperature | Bioclim 1 |
|  | Annual Mean Diurnal Range | Bioclim 2 |
|  | Isothermality | Bioclim 3 |
|  | Temperature Seasonality | Bioclim 4 |
|  | Max Temperature of Warmest Month | Bioclim 5 |
|  | Min Temperature of Coldest Month | Bioclim 6 |
|  | Annual Temperature Range | Bioclim 7 |
|  | Mean Temperature of Wettest Quarter | Bioclim 8 |
|  | Mean Temperature of Driest Quarter | Bioclim 9 |
|  | Mean Temperature of Warmest Quarter | Bioclim 10 |
|  | Mean Temperature of Coldest Quarter | Bioclim 11 |
|  | Annual Precipitation | Bioclim 12 |
|  | Precipitation of Wettest Month | Bioclim 13 |
|  | Precipitation of Driest Month | Bioclim 14 |
|  | Precipitation Seasonality | Bioclim 15 |
|  | Precipitation of Wettest Quarter | Bioclim 16 |
|  | Precipitation of Driest Quarter | Bioclim 17 |
|  | Precipitation of Warmest Quarter | Bioclim 18 |
|  | Precipitation of Coldest Quarter | Bioclim 19 |
| Land use variables  (Chini et al., 2014) | Percentage of cropland cover | Cropland |
|  | Percentage of primary land cover | Primary land |
|  | Percentage of secondary land cover | Secondary land |
|  | Percentage of pasture cover | Pasture |
|  | Percentage of urban land cover | Urban land |
| Digital Elevation Model (Jarvis et al. 2008) | Elevation | DEM |
