# Supplementary figures and images for: Climate Change Impact on Human-Rodent Interfaces: Modeling Junin Virus Reservoir Shifts
Source: Ecohealth. 2025 Jun 27;22(3):332–45. doi: 10.1007/s10393-025-01723-z (PMC12476445; doi:10.1007/s10393-025-01723-z)

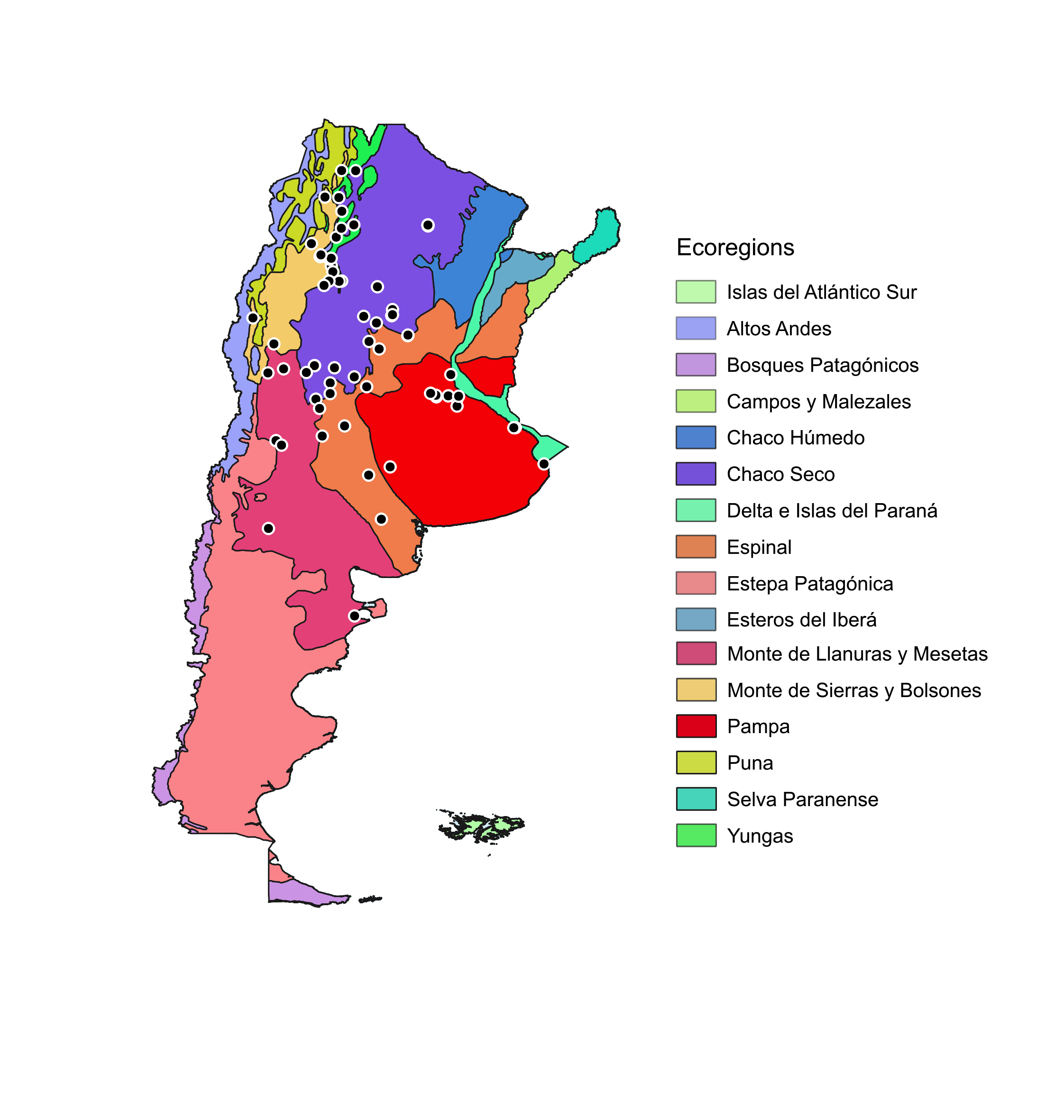

Supplement: Supplementary file 3 — Supplementary file3 (TIFF 521 KB) [file 10393_2025_1723_MOESM3_ESM.tiff]

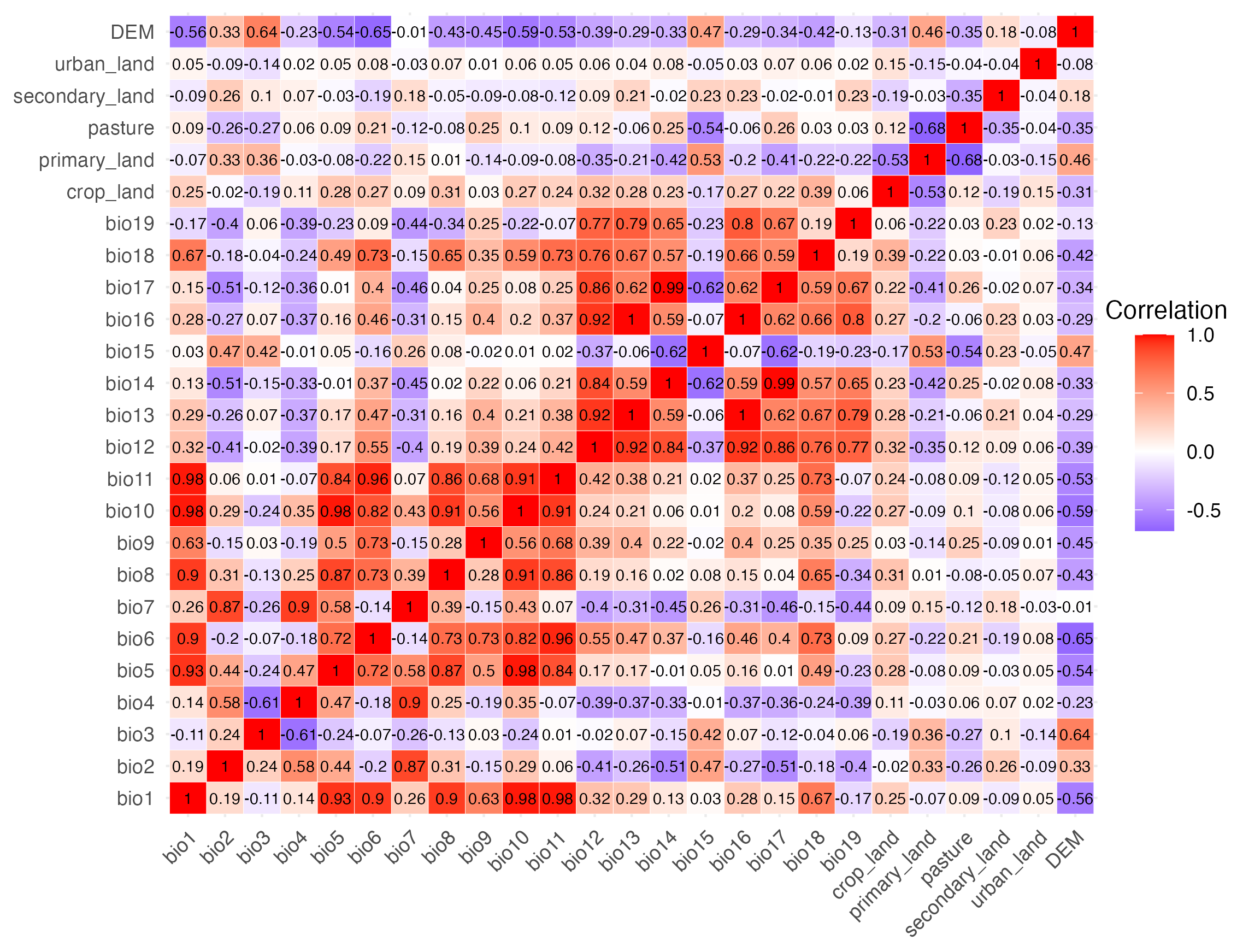

Supplement: Supplementary file 4 — Supplementary file4 (TIFF 1835 KB) [file 10393_2025_1723_MOESM4_ESM.tiff]

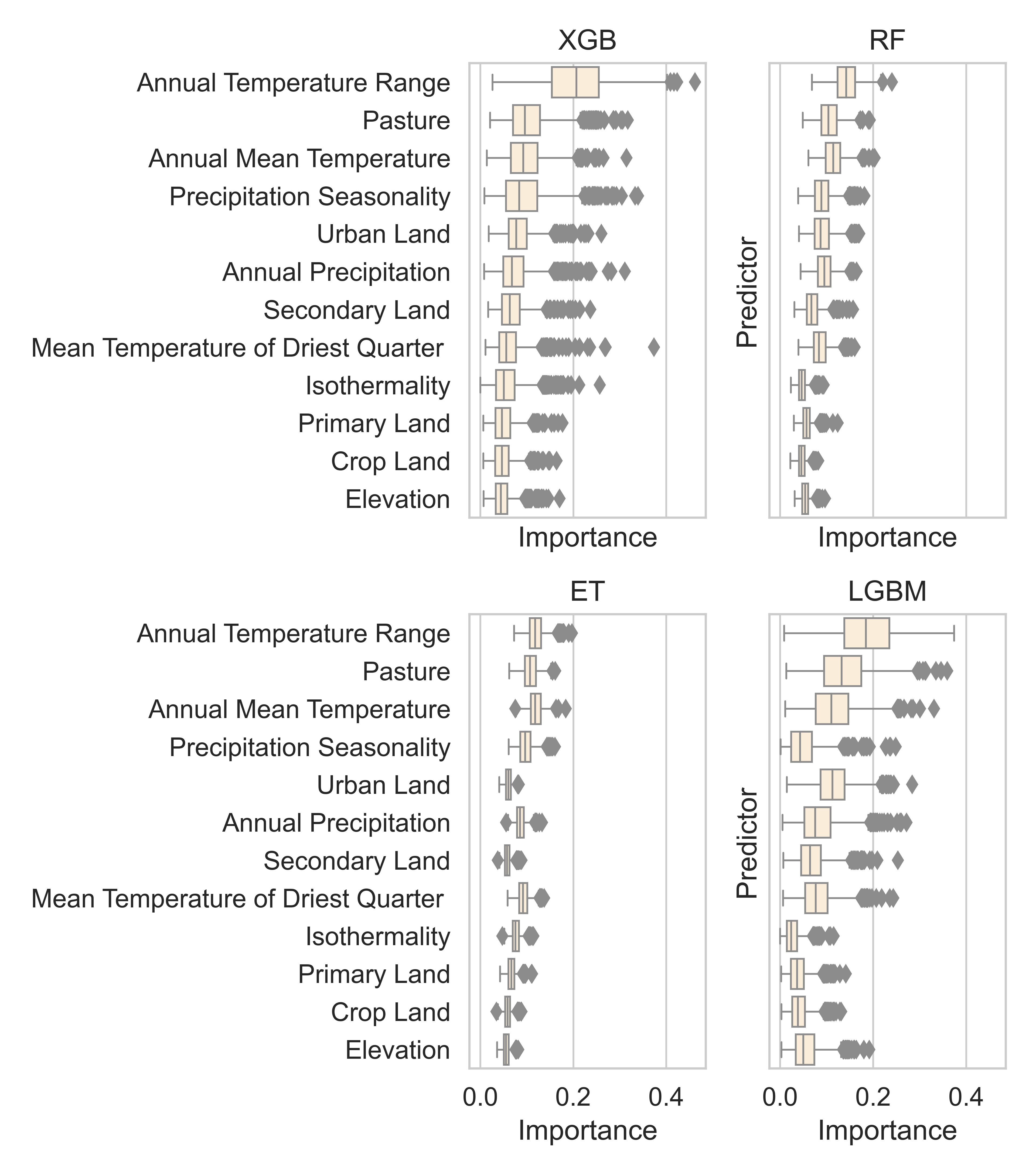

Supplement: Supplementary file 5 — Supplementary file5 (TIF 1803 KB) [file 10393_2025_1723_MOESM5_ESM.tif]

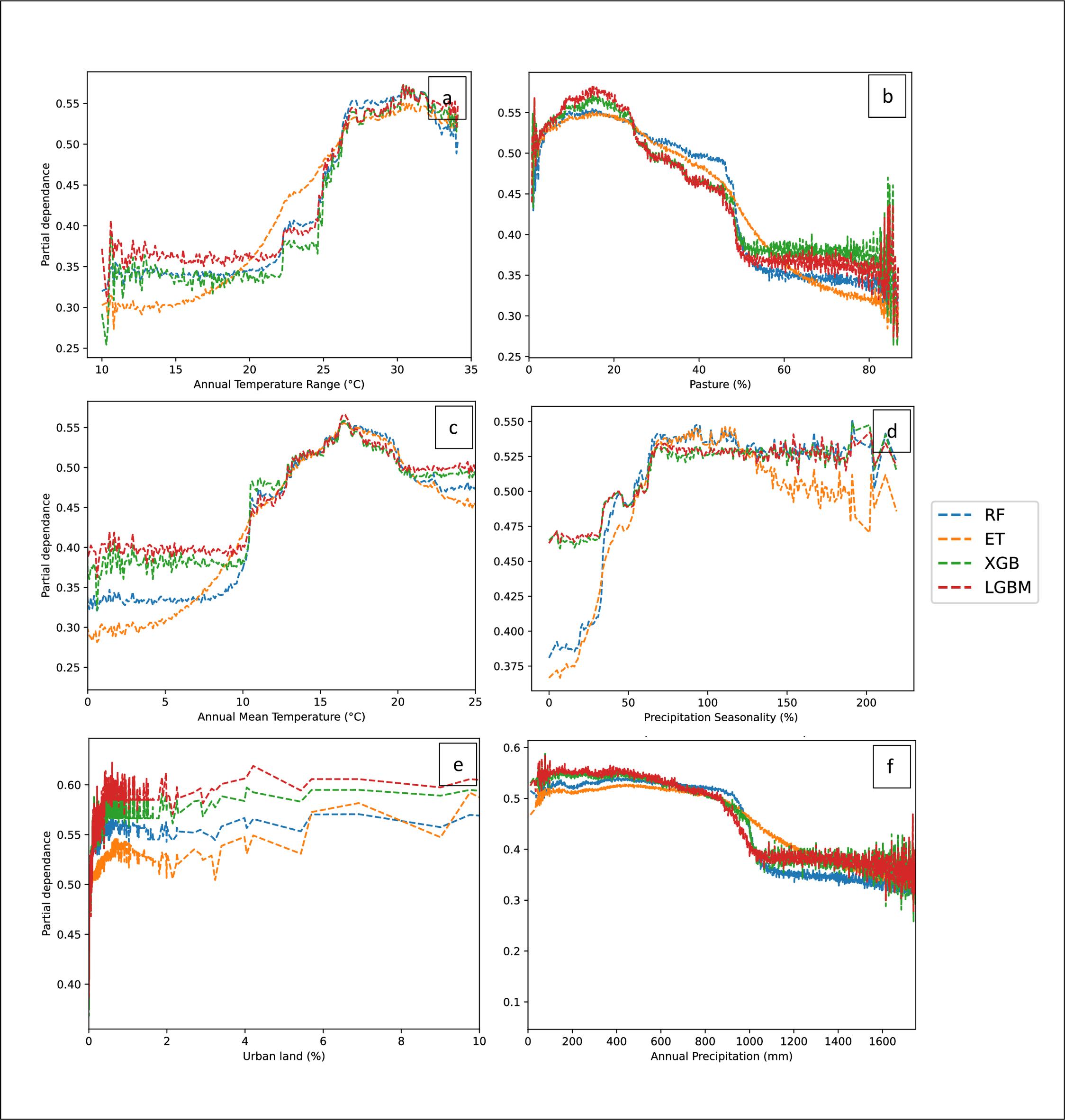

Supplement: Supplementary file 6 — Supplementary file6 (TIF 1575 KB) [file 10393_2025_1723_MOESM6_ESM.tif]

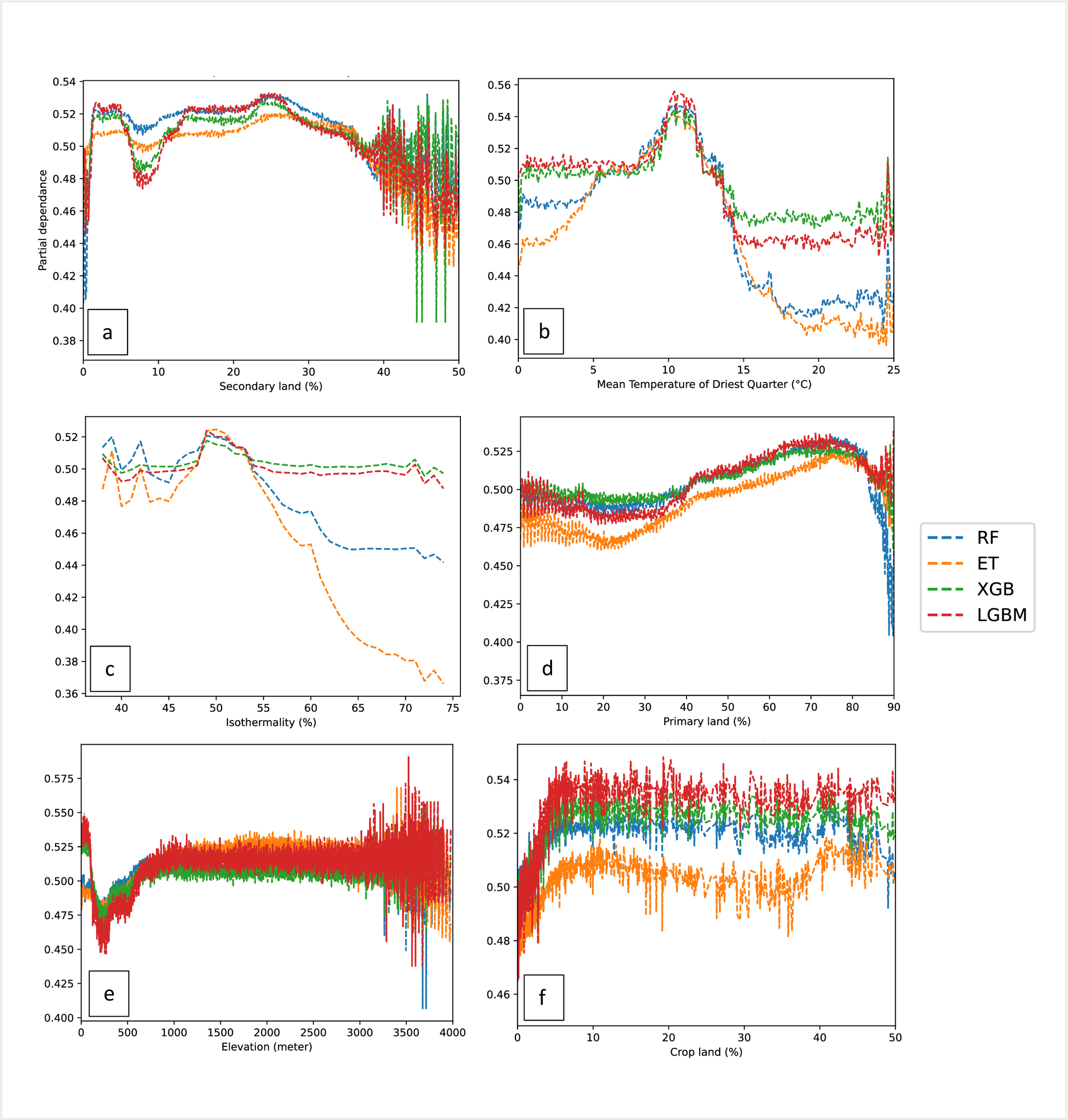

Supplement: Supplementary file 7 — Supplementary file7 (TIF 2054 KB) [file 10393_2025_1723_MOESM7_ESM.tif]

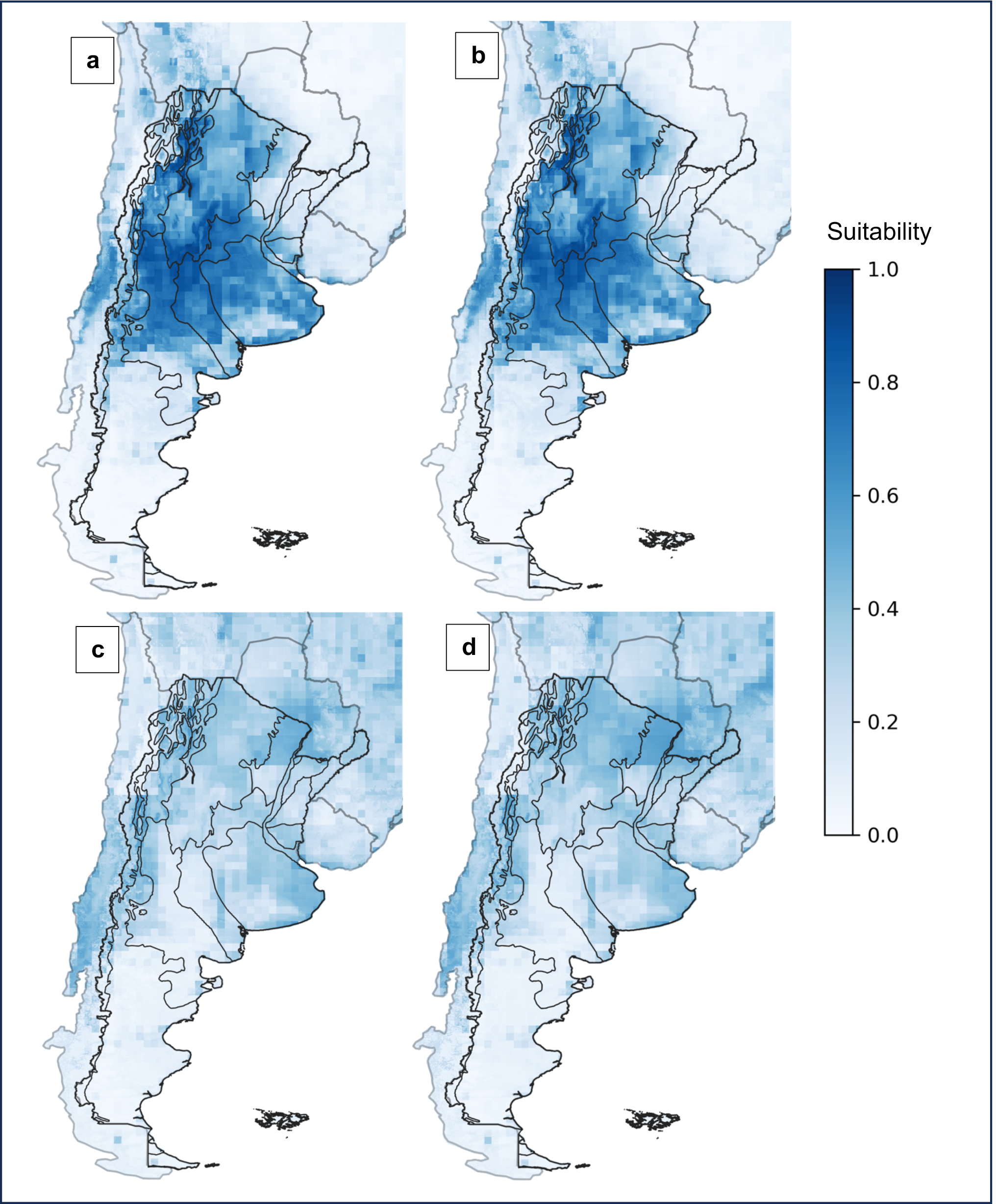

Supplement: Supplementary file 8 — Supplementary file8 (TIF 3382 KB) [file 10393_2025_1723_MOESM8_ESM.tif]
